# De-implementation of expensive blood saving measures in hip and knee arthroplasties: study protocol for the LISBOA-II cluster randomized trial

**DOI:** 10.1186/1748-5908-9-48

**Published:** 2014-04-23

**Authors:** Veronique MA Voorn, Perla J Marang-van de Mheen, Cynthia So-Osman, Ad A Kaptein, Anja van der Hout, M Elske van den Akker-van Marle, Ankie WMM Koopman-van Gemert, Albert Dahan, Rob GHH Nelissen, Thea PMM Vliet Vlieland, Leti van Bodegom-Vos

**Affiliations:** 1 Department of Medical Decision Making, Leiden University Medical Center, Albinusdreef 2, 2333 ZA, Leiden, The Netherlands; 2 Jon J van Rood, Netherlands Center for Clinical Transfusion Research, Sanquin Research, Plesmanlaan 1-A, 2333 BZ, Leiden, The Netherlands; 3 Department of Medical Psychology, Leiden University Medical Center, Albinusdreef 2, 2333 ZA, Leiden, The Netherlands; 4 Department of Anesthesiology, Albert Schweitzer Hospital, Albert Schweitzerplaats 25, 3318 AT, Dordrecht, The Netherlands; 5 Department of Anesthesiology, Leiden University Medical Center, Albinusdreef 2, 2333 ZA, Leiden, The Netherlands; 6 Department of Orthopaedics, Leiden University Medical Center, Albinusdreef 2, 2333 ZA, Leiden, The Netherlands

**Keywords:** Hip/knee arthroplasties, Blood transfusion, Patient blood management, Blood saving measures, De-implementation

## Abstract

**Background:**

Despite evidence that erythropoietin and intra- and postoperative blood salvage are expensive techniques considered to be non-cost-effective in primary elective total hip and knee arthroplasties in the Netherlands, Dutch medical professionals use them frequently to prevent the need for allogeneic transfusion. To actually change physicians’ practice, a tailored strategy aimed at barriers that hinder physicians in abandoning the use of erythropoietin and perioperative blood salvage was systematically developed. The study aims to examine the effectiveness, feasibility and costs of this tailored de-implementation strategy compared to a control strategy.

**Methods/Design:**

A cluster randomized controlled trial including an effect, process and economic evaluation will be conducted in a minimum of 20 Dutch hospitals. Randomisation takes place at hospital level. The hospitals in the intervention group will receive a tailored de-implementation strategy that consists of four components: interactive education, feedback in educational outreach visits, electronically sent reports on hospital performance (all aimed at orthopedic surgeons and anesthesiologists), and information letters or emails aimed at other involved professionals within the intervention hospital (transfusion committee, OR-personnel, pharmacists). The hospitals in the control group will receive a control strategy (*i.e*., passive dissemination of available evidence). Outcomes will be measured at patient level, using retrospective medical record review. This will be done in all hospitals at baseline and after completion of the intervention period. The primary outcome of the effect evaluation is the percentage of patients undergoing primary elective total hip or knee arthroplasty in which erythropoietin or perioperative blood salvage is applied. The actual exposure to the tailored strategy and users’ experiences will be assessed in the process evaluation. In the economic evaluation, the costs of the tailored strategy and the control strategy in relation to the difference in their effectiveness will be compared.

**Discussion:**

This study will show whether a systematically developed tailored strategy is more effective for de-implementation of non-cost-effective blood saving measures than the control strategy. This knowledge can be used in national and international initiatives to make healthcare more efficient. It also provides more generalized knowledge regarding de-implementation strategies.

**Trial registration:**

This trial is registered at the Dutch Trial Register NTR4044.

## Background

Total joint replacement surgery such as total hip arthroplasty (THA) and total knee arthroplasty (TKA) is associated with intra- and postoperative blood loss leading to postoperative anemia. This can be subsequently treated with allogeneic blood transfusion [1,2]. Yet, allogeneic blood transfusions carry the risk of infections and non-infectious transfusion reactions [3]. Therefore, different types of blood saving measures (BSMs) have been developed to reduce blood loss or to increase cell mass to avoid allogeneic transfusions [4].

Many studies on the effectiveness of the frequently used BSMs erythropoietin (EPO) and intra- and postoperative drainage and re-infusion of autologous blood (in short: perioperative blood salvage) in orthopedic surgery have been performed. Reviews and meta-analyses showed that EPO and perioperative blood salvage reduce transfusions. However, the included studies had several limitations such as a retrospective design, small patient numbers and poor methodologically quality leading to bias in favor of EPO and perioperative blood salvage [1,5-10]. When the costs of these techniques are considered, the use of EPO and perioperative blood salvage becomes controversial [8,11-17]. A large multicenter Randomized Controlled Trial (RCT) was recently performed to test the effectiveness and cost-effectiveness of EPO and perioperative blood salvage in elective THA and TKA [18,19]. It was shown that perioperative blood salvage in primary THA and TKA neither resulted in a decreased mean number of allogeneic blood units nor in a decrease in the proportion of transfused patients, and was more expensive due to the costs of the device and a prolonged hospital stay. EPO showed a significant decrease in the mean number of allogeneic blood units and proportion of transfused patients, but the costs of this technique were considered too high. It was thus concluded that EPO and perioperative blood salvage were not cost-effective in primary elective THA and TKA. For the use of EPO and perioperative blood salvage in revision THA and TKA no conclusions about the (cost-) effectiveness could be drawn [18,19]. These results are in line with recent literature. A number of trials that were not included in the currently available meta-analyses show that perioperative blood salvage is not superior to a regular drain or no drain in THA or TKA [20-24], other studies concerning the costs of EPO also doubt the cost-effectiveness in orthopedic surgery [11,14].

Despite the evidence, medical professionals keep using these BSMs in daily practice. Over 85% of Dutch hospitals frequently use EPO, perioperative blood salvage, or a combination of these in elective orthopaedic surgery [25]. This leads to unnecessary healthcare costs. So, to improve the efficiency of care delivery, a strategy is needed aimed at barriers and facilitators to stop using these non-cost-effective BSMs (de-implementation strategy) [26-29]. In the ‘Leiden Implementation Study of BlOod management in hip and knee Arthroplasties’ (LISBOA I) problem analysis study [30], such a strategy was developed in accordance with the implementation model of Grol [31]. This model, as with other theories of change, emphasizes that changes in current practice can only take place after the current barriers and facilitators for change have been identified and targeted. Therefore, prior inventory of barriers and facilitators incorporated in a tailored strategy can reduce the number of costly trials evaluating different implementation strategies [31-33]. The current study will test the hypothesis that the developed strategy is more effective for de-implementation of EPO and perioperative blood salvage in elective primary THA and TKA in comparison with a control strategy (*i.e*., passive dissemination of evidence).

### Objective

The ‘Leiden Implementation Study of BlOod management in hip and knee Arthroplasties, part two’ (LISBOA-II) aims to assess the effectiveness, feasibility and costs of a systematically developed tailored strategy for de-implementation of EPO and perioperative blood salvage in primary elective THA and TKA [30] compared to a control strategy in a cluster randomized trial.

## Methods

### Study design

A cluster randomized controlled trial including an effect-, process- and economic evaluation will be conducted in a minimum of 20 hospitals in the Netherlands using EPO and/or perioperative blood salvage in THA and TKA. Per hospital a representative orthopedic surgeon will be invited to participate in the study (see Additional file 1 for CONSORT checklist); consent of hospitals willing to participate will be gathered according to local hospital regulations. To prevent contamination bias, randomisation will take place at the hospital level stratified by geographic location of the hospitals. Randomisation will be performed by an independent researcher using a computer generated randomisation table concealed in a sealed envelope. The randomisation result will be revealed to the investigators and participating hospitals after the baseline measurement on effect outcomes takes place.

This trial compares:

1. The tailored strategy to de-implement use of EPO and perioperative blood salvage, and

2. A control strategy.

**Figure 1:**
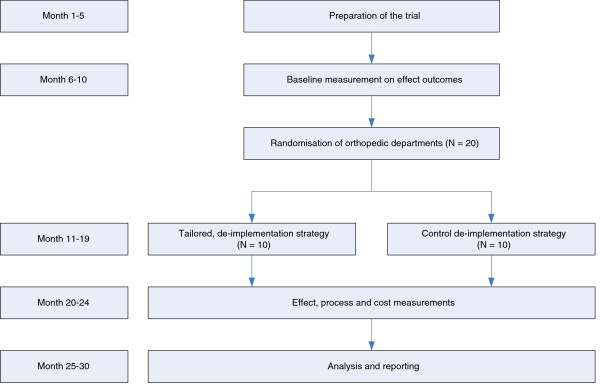
Flow-chart study design.

See Figure 1 for a flow-chart of the study design.

### Intervention

#### Tailored de-implementation strategy

The tailored de-implementation strategy in intervention hospitals is aimed at the barriers for de-implementation of non-cost-effective BSMs as identified in our problem analysis study, in which representative samples of orthopedic surgeons and anesthesiologists participated [30,34]. To ensure that we identified all relevant barriers, we used the Theoretical Domains Framework (TDF) [35-37]. The TDF includes 12 different domains derived from a large number of health psychology theories and their theoretical constructs. The main barriers to stop using non-cost-effective BSMs in elective orthopedic surgery were perceived by physicians within the following domains of the TDF:

1. Knowledge: lack of alternatives, lack of interest to gain additional information about stopping EPO and perioperative blood salvage.

2. Motivation & goals: lack of interest to save money for the society/ hospital, lack of benefit for the delivery of care.

3. Beliefs about consequences: pressure of suppliers to use BSMs, concerns about losing experience with the use of BSMs, concerns about the safety of patients when BSMs are stopped.

4. Social influences: the impact of blood management policy of other medical specialties/ blood transfusion committee, lack of influence of individual physician on blood management policy.

Barriers for EPO and perioperative blood salvage are largely similar and found within the same domains. Some barriers are more relevant for de-implementation of perioperative blood salvage (for example, concerns about losing experience with the use of BSMs) than for de-implementation of EPO. However, due to the large extent of overlap of barriers and the similar target groups, we developed a combined tailored strategy.

The developed tailored de-implementation strategy consists of four components carried out in a period of nine months. Every component targets one or more domains on which barriers have been identified [35].

1. Interactive education for orthopedic surgeons and anesthesiologists with a single visit in intervention month 1 (to target the domain: motivation & goals).

2. Feedback in educational outreach visits for orthopedic surgeons and anesthesiologists with a single visit in intervention months 5/6 (to target the domain: beliefs about consequences).

3. Dissemination of reports on hospital performance (BSM use and transfusion percentage) and comparison with hospitals that do not use EPO or perioperative blood salvage, *e.g*., ‘best practices,’ to orthopedic surgeons and anesthesiologists with two electronic newsletters sent in intervention months 4 to 6 and 7 to 9 (to target the domain: social influences).

4. Email with available evidence to other involved professionals, *e.g*., transfusion committee, OR-personnel, pharmacists with a single newsletter sent in intervention months 1 or 2 (to target the domains: knowledge, motivation and goals, and beliefs about consequences).

**Figure 2:**
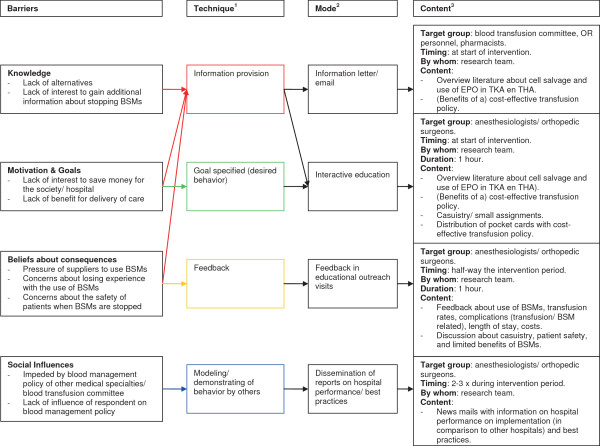
**De-implementation strategy. **^1^Technique: the behavior change technique used to overcome the identified barrier. ^2^Mode: the way the technique will be delivered. ^3^Content: what will be delivered.

For the complete de-implementation strategy, see Figure 2.

#### Control strategy

The control strategy consists of the passive dissemination of evidence via publication in scientific journals indexed for PubMed. No further actions to make the control hospitals aware of the published evidence will be undertaken. After the post-intervention measurement period, we will offer the control hospitals the possibility to have the interactive education as described for the intervention hospitals and a report on their hospital performance in comparison with best practices.

#### Study population

All types of hospitals (university hospitals, teaching hospitals, general hospitals and private clinics) that frequently use BSMs [25] will be invited to participate in this study. Further, we have previously shown that blood transfusion committees, hospital boards, patients, and other stakeholders are involved in blood management, but do not make decisions regarding the use of BSMs in THA and TKA [34]. Therefore, the de-implementation strategy will primarily be focused at orthopedic surgeons and anesthesiologists in the participating hospitals, with one strategy-component aimed at other involved professionals.

See Table 1 for in- and exclusion criteria for the participating hospitals and patients.

**Table 1:** In- and exclusion criteria for participating hospitals and patients

**Inclusion criteria**	**Exclusion criteria**
Participating hospitals	
• Hospitals using EPO and/or blood salvage in patients undergoing primary elective THA or TKA on a regular basis (more frequently than in exceptional cases)	• Hospitals considering abandoning the use of EPO or blood salvage on their own initiative
• Hospitals performing at least 50 THA and/or TKA on average per 5 months	• Hospitals participating in trials that interfere with the use or the discontinuation of EPO or blood salvage
	• Hospitals employing the same group of orthopaedic surgeons or anaesthesiologists as a previously included hospital
Patients	
• Patients scheduled for primary elective THA or TKA	• Bilateral surgery
• Age >18 years	• Patients with a malignancy (except skin cancer or cured cancers)
	• A serious disorder of the coronary, peripheral and/or carotid arteries, a recent myocardial infarction or CVA (past 6 months)
	• Untreated hypertension (diastolic BP >95 mmHg)
	• Patients with a pregnancy
	• Patients with a coagulation disorder
	• Patients refusing or with a contraindication for allogeneic blood transfusions
	• Patients with untreated anaemia Hb <10 g/dl

#### Evaluation and outcome measures

1. Effect evaluation

The effect of the tailored strategy will be compared with the control strategy before and after carrying out the strategy. Outcomes will be measured at patient level, using retrospective medical record review at least three weeks postoperative with standardized registration forms. Measurement periods last for five months. Within each month, medical records of at least 10 consecutively treated patients will be reviewed in each participating hospital, with a maximum of 20 patients per month (depending on the number of patients treated within that month).

#### Primary outcome

The primary outcome is the % of patients undergoing primary elective total THA or TKA in which EPO or perioperative blood salvage is applied.

#### Secondary outcomes

Secondary outcomes are the patient outcomes of the surgery including: Post-operative hemoglobin (Hb) level, length of hospital stay and number of allogeneic red blood cell transfusions. Adverse events will also be registered: reactions on EPO use, transfusion reactions due to the use of perioperative blood salvage, transfusion reactions due to allogeneic transfusions and complications registered in patients’ medical records.

Other parameters measured in this study are patient characteristics (age, sex, BMI, ASA-classification, and pre-operative Hb), techniques used during the surgical procedure (type of anesthesia, use of tourniquet in TKA, surgical approach, use of other BSMs, use of drains) and postoperative care (postoperative blood loss, re-infusion of salvaged blood, type and length of postoperative anticoagulation).

2. Process evaluation

A process evaluation will be performed to assess the feasibility of the de-implementation strategy in comparison with the control strategy. Such an evaluation gives insight into the mechanisms and processes responsible for the effect of the de-implementation strategy and the control strategy [38]. The actual ‘exposure’ of clinicians to the elements of the de-implementation strategy, together with their experience with these elements, may influence the final results. At the end of the study period, experiences of clinicians with the elements of the de-implementation strategy will be measured using questionnaires, to further improve the de-implementation strategy for future use (if necessary). In these questionnaires, we will also ask about the presence and their awareness of barriers for behavior change.

Other local, non-study-related changes such as changes in staff, changes in blood management, changes in surgical techniques and local initiatives to optimize THA and TKA care will be registered in both study arms. See also Table 2.

3. Economic evaluation

**Table 2:** Overview of measurements

	**Baseline measurements**		**Intervention period**		**Post-intervention measurements**	
	**Intervention**	**Control**	**Intervention**	**Control**	**Intervention**	**Control**
**On physician level**						
Barrier questionnaire					x	x
Process evaluation			x	x	x	
Resource use					x	x
**On patient level (through retrospective chart review)**						
Primary and secondary outcomes, complications and adverse events	x	x	x		x	x
Patient characteristics	x	x	x		x	x
Techniques during surgical procedure	x	x	x		x	x
Postoperative care	x	x	x		x	x

The economic evaluation will compare the costs of both de-implementation strategies in relation to their difference in effects. The analyses will not be performed separately for the de-implementation of EPO and perioperative blood salvage since it is impossible to determine which costs of the de-implementation strategy are exclusively made for the de-implementation of EPO and for the de-implementation of perioperative blood salvage. The economic evaluation will be performed from a healthcare perspective. No discounting will be applied due to the short time frame of the study.

The implementation costs concern the cost of execution of the de-implementation strategy [39], which consist of material costs (*e.g*., education material, information letter), and personnel cost (*e.g*., hours for the team of investigators conducting the strategy, hours of orthopedic surgeons and anesthesiologists attending the strategy-related activities). Resource use will be measured by questionnaires to the clinicians involved. For the valuation of the resource use, market prices (material) and wages including holiday allowance and social charges (personnel costs) are used [40].

Table 2 provides an overview of all measurements.

### Statistical analysis

All data will be entered and stored in an electronic database. Descriptive statistics include frequencies, percentages, medians, means and SDs. Hospital and patient characteristics of study hospitals will be compared using t-tests and non-parametric tests (Mann-Whitney U test) for continuous variables and χ^2^-test for proportions. The overall effect of the intervention will be evaluated by comparing the average outcome in the control hospitals with the average outcome in the intervention hospitals. The effects on the percentage of patients receiving a THA or TKA in which BSMs (stratified to the % patients with EPO and % patients with perioperative blood salvage) are applied will be adjusted for clustering of patients in hospitals. Therefore, multilevel logistic regression analysis will be performed. Analyses will be based on the intention to treat principle, meaning that all participating hospitals will be included in the study arm (control or intervention) to which they are originally assigned, regardless of whether they participated in the components of the tailored strategy.

### Sample size

We expect to detect an absolute difference of at least 20% in BSM use between the group receiving the de-implementation strategy and the control group. We assume that frequent BSM use, as assessed in the Dutch survey [25], means that BSMs are applied in 50% of the patients. To detect a difference of 20% (from 50% to 30%), with alpha 0.05, a two-sided testing and power of 80%, an intra-cluster-correlation coefficient of 0.08, 50 patients per hospital and 20 hospitals are needed (total of 1,000 patients). Given the 70% of hospitals in The Netherlands frequently applying BSMs [25], this means that 69 hospitals are eligible for the present study. Since the average hospital performs about 550 total hip and knee arthroplasties per year, it is feasible to include 20 hospitals and at least 50 patients per hospital in 5 months, before and after the intervention period.

### Ethical approval

The Medical Ethical Committee of the Leiden University Medical Center decided that ethical approval was not required under Dutch National law for this type of study (CME 13/132). The gathering of patient data will be conducted in compliance with the Good Clinical Practices protocol and Declaration of Helsinki principles [41].

### Trial status

The LISBOA II study started in March 2013. The preparation of the study components and the recruitment of hospitals to participate in the study were completed in September 2013. The baseline data collection in all hospitals was performed from September 2013 until January 2014. Currently (March 2014), the intervention period is ongoing.

## Discussion

The goal of this study is to test a tailored strategy to let physicians stop using EPO and perioperative blood salvage in primary elective THA and TKA, *i.e*., the de-implementation of non-cost-effective BSMs. This study is the next step following a RCT on EPO and perioperative blood salvage as transfusion alternatives in THA and TKA using a restrictive transfusion policy, showing that use of these BSMs is not cost-effective [18,19], and a study in which a tailored de-implementation strategy was systematically developed [25,30,34]. Given the large number of THA and TKA performed annually in the Netherlands and worldwide, de-implementation of non-cost effective BSMs contributes to more efficient healthcare.

A strength of this study is that it is one of the first studies that assesses the effect of a de-implementation strategy. The study results will thus lead to generalizable knowledge regarding de-implementation strategies of non-cost-effective interventions and how this differs from strategies for implementation. This knowledge is useful to contain healthcare spending and optimize outcomes [26,27].

A possible limitation of the study is the awareness of the study purpose among physicians within the control group. During the recruitment of hospitals for participation in our study, hospitals cannot be blinded to the aim of this study. Physicians want to know the study goal before giving approval for participation in a study. As a consequence, physicians of control hospitals are actively made aware of the fact that they deliver non-efficient care and thereby can make changes in their blood management policy. This does not resemble ‘standard practice’ in hospitals not participating in a study and may lead to a smaller difference in the effect between the intervention and control hospitals. We will try to limit this awareness by asking the study coordinators of each participating hospital not to inform their staff members (orthopedic surgeons and anesthesiologists) about the study.

A second limitation is bias as a result of local initiatives to optimize care for THA and TKA during the intervention period. For example, the implementation of ‘fast track’ or ‘joint care’ programs for THA and TKA may lead to abandoning perioperative blood salvage because of logistic reasons. Therefore, information on local, non-study related changes will be additionally inquired during the process evaluation.

Our study will not only demonstrate whether a tailored strategy to de-implement BSMs is effective, feasible and cost-effective compared to the control strategy, but will also contribute to general knowledge regarding differences between de-implementation and implementation strategies. Little is known about strategies to effectively de-implement common practices, for instance, whether de-implementation strategies should also be constructed following the same theoretical models and frameworks as implementation. It is likely that it is far more attractive for clinicians to implement something new than to de-implement something expensive or ineffective [26,27]. Our study will thus not only assess whether a tailored strategy to de-implement BSMs is effective, feasible and cost-effective compared to the control strategy, but will also contribute to general knowledge regarding differences between de-implementation and implementation strategies.

## Abbreviations

BSMs: Blood saving measures; EPO: Erythropoietin; THA: Total hip arthroplasty; TKA: Total knee arthroplasty; RCT: Randomized controlled trial; LISBOA: Leiden implementation study of blood transfusion management in hip and knee arthroplasties; TDF: Theoretical domains framework; CME: Medical ethics committee (in Dutch: Commissie medische ethiek); SDs: Standard deviations; BMI: Body mass index; ASA classification: American society of anesthesiologists classification; Hb: Hemoglobin.

## Competing interests

The authors declare that they have no competing interests.

## Authors’ contributions

LB, PM, VV and TV designed the study. VV and AH will carry out the study. LB, PM and VV coordinate the study. VV, LB and PM drafted the manuscript. All authors have critically read and modified the study protocol and previous drafts of the manuscript, and have approved the final version.

## Supplementary Material

Additional file 1 CONSORT checklists. Click here for file
